# Enhanced Electrochemical Properties of Gel Polymer Electrolyte with Hybrid Copolymer of Organic Palygorskite and Methyl Methacrylate

**DOI:** 10.3390/ma11101814

**Published:** 2018-09-24

**Authors:** Lanlan Tian, Lian Xiong, Xuefang Chen, Haijun Guo, Hairong Zhang, Xinde Chen

**Affiliations:** 1 Guangzhou Institute of Energy Conversion, Chinese Academy of Sciences, Guangzhou 510640, China; plshm126@126.com (L.T.); xionglian@ms.giec.ac.cn (L.X.); chenxue_228@126.com (X.C.); guohaijun6621@163.com (H.G.); 2 Key Laboratory of Renewable Energy, Chinese Academy of Sciences, Guangzhou 510640, China; 3 Guangdong Provincial Key Laboratory of New and Renewable Energy Research and Development, Chinese Academy of Sciences, Guangzhou 510640, China; 4 R&D Center of Xuyi Attapulgite Applied Technology, Guangzhou Institute of Energy Conversion, Chinese Academy of Sciences, Xuyi 211700, China; 5 University of Chinese Academy of Sciences, Beijing 100049, China

**Keywords:** lithium polymer battery, poly (organic palygorskite-co-methyl methacrylate), microporous polymer electrolyte

## Abstract

Gel polymer electrolyte (GPE) is widely considered as a promising safe lithium-ion battery material compared to conventional organic liquid electrolyte, which is linked to a greater risk of corrosive liquid leakage, spontaneous combustion, and explosion. GPE contains polymers, lithium salts, and liquid electrolyte, and inorganic nanoparticles are often used as fillers to improve electrochemical performance. However, such composite polymer electrolytes are usually prepared by means of blending, which can impact on the compatibility between the polymer and filler. In this study, the hybrid copolymer poly (organic palygorskite-co-methyl methacrylate) (poly(OPal-MMA)) is synthesized using organic palygorskite (OPal) and MMA as raw materials. The poly(OPal-MMA) gel electrolyte exhibits an ionic conductivity of 2.94 × 10^−3^ S/cm at 30 °C. The Li/poly(OPal-MMA) electrolyte/LiFePO_4_ cell shows a wide electrochemical window (approximately 4.7 V), high discharge capacity (146.36 mAh/g), and a low capacity-decay rate (0.02%/cycle).

## 1. Introduction

Rechargeable lithium batteries (LIBs) have long been regarded as the most promising energy storage technology for various portable electronics, electric cars, and energy storage systems [1,2]. Generally, the use of liquid organic solvents as an electrolyte in lithium batteries raises safety concerns, owing to the possible leakage of corrosive electrolytes and the risk of spontaneous combustion and explosion when batteries are subjected to high temperatures and/or violent impacts [3]. Additionally, liquid electrolytes can react with the lithium anode metal to form “dead lithium”, which limits liquid electrolyte use in LIBs [4]. In order to obtain stable and secure electrolytes, a great deal of research work has been done on solid electrolytes, such as solid polymer electrolytes [5]. However, solid electrolytes have some disadvantages, such as low ionic conductivity (<10^−5^ S/cm), low permeability, and high interfacial resistance, which restrict their industrial applications.

Polymer matrices can absorb a large amount of liquid electrolyte solution to form gel polymer electrolytes (GPEs). GPEs exhibit high ionic conductivity, good interface stability, and a long service life [5,6,7,8,9]. In particular, poly (methyl methacrylate) (PMMA)-based electrolyte is regarded as a promising material because PMMA has good chemical stability, is a low-cost raw material, and can reduce the interfacial resistance between the polymer electrolyte and the electrodes [10,11,12,13,14,15,16]. However, the major challenge of a large-scale application of PMMA is its low thermal stability and low ionic conductivity. In order to increase the ionic conductivity of polymer electrolytes, many organic and inorganic modifiers, such as polyvinylidene fluoride (PVDF) [16], poly (vinylidene fluoride-hexafluoropropylene) (poly (VdF-HFP)) [17], poly (vinyl chloride) (PVC) [18], SiO_2_ [19], TiO_2_ [20], Al_2_O_3_ [21], and clay, are added into the polymer matrices. Clays such as montmorillonite and palygorskite (Pal) have several distinct advantages as fillers, namely: A high aspect ratio (∼1000), high cation-exchange capacity, large specific surface area, and appropriate interlayer charge [22,23,24]. Pal is a member of a group of clay minerals comprised of magnesium aluminosilicate [25]. It has a unique, natural fibrous or rod-like, structure and many hydroxyl groups on its surface. Pal has good compatibility with polymer matrices after suitable surface treatments [25,26,27]. Pal has been widely used as the polymer enforcement dispersed in polymer matrices [28]. Chen et al. [27] reported that Pal can significantly reinforce the mechanical properties and thermal stability of PMMA after its surface has been treated with an organic reagent. Yao et al. [26] found that Pal can not only improve the stiffness and toughness of a PVDF-based polymer electrolyte, but can also enhance the transference number of Li^+^.

Generally, most Pal/polymer composite materials are prepared by traditional physical blending, which would impact their compatibility, making it difficult to see their beneficial characteristics, especially the nanoscale effects of Pal, as well as limiting their application.

In this work, poly(OPal-methyl methacrylate) (poly(OPal-MMA)) has been copolymerized by solution polymerization, using OPal and MMA as raw materials. A coin-type cell was assembled using poly(OPal-MMA) as the separator, lithium as the anode, and lithium iron phosphate (LiFePO_4_) as the cathode, and the electrochemical characteristics of poly(OPal-MMA), such as its ionic conductivity, electrochemical window, and cycling performance, were evaluated.

## 2. Materials and Methods

### 2.1. Materials

Methyl methacrylate (MMA), anhydrous ethanol, 1-methyl-2-pyrrolidinon (NMP), potassium peroxydisulfate (K_2_S_8_O_4_), sodium bisulfite (NaHSO_3_), N,N-Dimethylformamide (DMF), and acetone were of an analytical grade. Organic palygorskite (OPal) modified by dimethyl diallyl ammonium chloride was purchased from Zhongke New Energy Co., Ltd. (Huai’an, China). Lithium iron phosphate (LiFePO_4_) powder and carbon black were purchased from Beijing HWRK Chem Co., Ltd. (Beijing, China). Binder (polyvinylidene fluoride PVDF), lithium tablets, and aluminum foil were purchased from Mingruixiang Automation Equipment Co., Ltd. (Shenzhen, China).

### 2.2. Preparation of Poly(OPal-MMA)

**Scheme 1:**
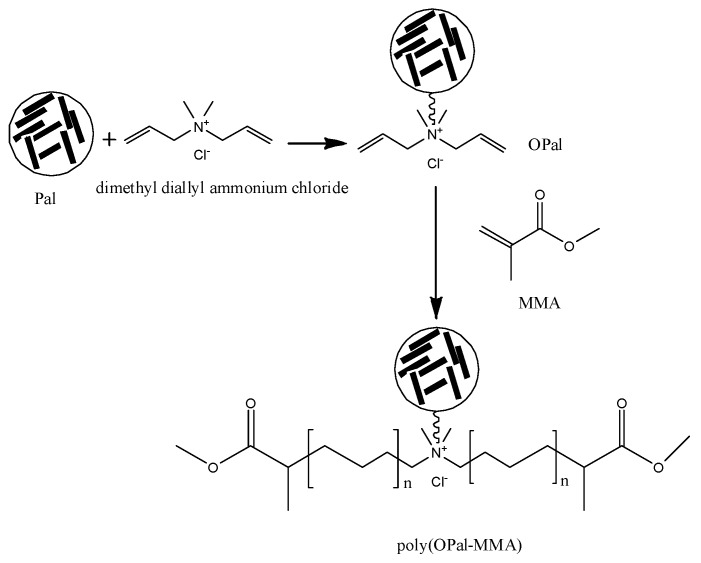
The reaction mechanism of poly(OPal-MMA).

The reaction mechanism of the poly(OPal-MMA) is shown in Scheme 1. A mixture of reaction solvents (deionized water/anhydrous ethanol; 2/1; *v*/*v*) and a given amount of OPal were added to a flask, and the flask was then preheated to 60 °C. Nitrogen gas was bubbled into the solution for 30 min, and a certain amount of K_2_S_4_O_8_ (>99%) and Na_2_SO_3_ (>99%) was then added, followed by MMA (99%) added drop by drop (one drop per second). After 5 h, the reaction temperature was raised to 80 °C for 1 h. The poly(OPal-MMA) was then filtrated, washed and purified, and then dried at 60 °C for 12 h in a vacuum oven. For the comparative experiments, a blend of PMMA and OPal (PMMA/OPal blend) was prepared by evenly mixing 95 g of MMA with 5 g of OPal, using mechanical agitation for 2 h.

### 2.3. Preparation of the Poly(OPal-MMA) Membranes

A given amount of polymer was dissolved in a mixture of organic solvent (dimethylformamide (DMF)/acetone; 1/15; *v*/*v*). The solution was uniformly coated on a glass pane, and then the coated pane was put into deionized water in order to remove the organic solvent. Then, the polymer membrane was dried at 80 °C for 24 h in a vacuum drying oven. The polymer membrane was then cut into a disk with a radius of 9 mm.

### 2.4. Characterization of Poly(OPal-MMA)

The micromorphology of poly(OPal-MMA) was observed using a scanning electron microscope (SEM) (Hitachi, S-4800 FESEM, Tokyo, Japan). The sample was coated with platinum to reduce the charging effects before being examined. The infrared spectrum of the sample was recorded on a Fourier transform infrared spectrophotometer (Bruker, TENSOR 27, Karlsruhe, Germany) from 4000 to 450 cm^−1^, using potassium bromide (KBr) pellets. The thermal behavior of the polymer was studied using Q50 (TA Instruments, New Castle, DE, USA) from 40 °C to 900 °C, with a linear heating rate of 10 °C/min in a nitrogen atmosphere.

### 2.5. Electrochemical Characteristics Analysis

The poly(OPal-MMA) membrane was submersed in a 1 mol/L LiClO_4_ electrolyte solution (propylene carbonate/ethylene carbonate; 1/1; *v*/*v*) at 25 °C in a glove box. Then the excess solution on the surface of the membrane was absorbed with filter paper, and the electrolyte uptake (EU) was calculated by the following Equation [8]:

(1) $$Electrolyte\;uptake\;(\%)=\frac{W_{1}-W_{0}}{W_{0}}\times 100\%\;$$

where *W*_1_ is the weight of membrane after absorbing the liquid electrolyte, and *W*_0_ is the weight of the membrane before absorbing the liquid electrolyte.

An electrochemical cell was assembled by sandwiching poly(OPal-MMA) electrolytes between two stainless steel blocking electrodes (surface area of 2.54 cm^2^), and the cell was then sealed. The ionic conductivity was measured using the alternating current (AC) impedance spectra of the stainless steel (SS)/polymer electrolyte/SS at 30 °C. The measurement was carried out over a frequency range of 0.1 Hz to 100 KHz, and the amplitude was 5 mV. The ionic conductivity (*σ*) was calculated using the following Equation [16,29]:

(2) $$\sigma =\frac{L}{R_{b}\times A}\;$$

where *L* is the thickness (cm), *R_b_* is the bulk resistance (ohm), and *A* is the effective area (2.54 cm^2^) of the polymer electrolyte.

The electrochemical window of the poly(OPal-MMA) electrolyte was studied using linear sweep voltammetry (LSV) at a scan rate of 0.5 mV·s^−1^ with an electrochemical cell. The cell was assembled in an argon-filled glove box, which consisted of an SS working electrode and lithium metal (reference electrode), as well as a counter electrode in a CR2032 coin-type cell.

The cathode was made with a mixture composed of 80 wt % LiFePO_4_ powder, 10 wt % PVDF, and 10 wt % carbon black, all of which were mixed with N-methylpyrrolidone (NMP). The slurries were spread onto aluminum foil and dried in a vacuum oven at 80 °C for 24 h. The LiFePO_4_ electrode (about 1.56 mg/cm^2^) was assembled as the positive electrolyte versus the Li metal as the negative electrode in a CR2032 coin-type cell. The Li/poly(OPal-MMA) electrolyte/LiFePO_4_ cell was mounted in a glove box filled with argon. The cell charge–discharge test was carried out between 2.8~4.0 V on a Neware battery tester (Shenzhen Neware Electronics Co. Ltd., Shenzhen, China) at room temperature (25 °C).

## 3. Results

### 3.1. Physical Characterization of Poly(OPal-MMA) Polymers

**Figure 1:**
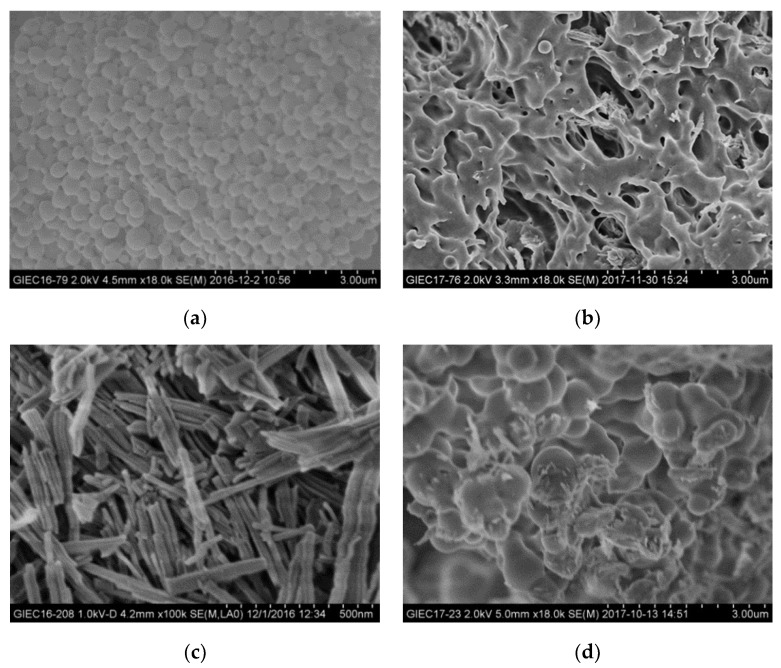
SEM images of poly (methyl methacrylate) (PMMA), poly(OPal-MMA), palygorskite (Pal), and the PMMA/OPal blend. (**a**) PMMA; (**b**) poly(OPal-MMA) (5 wt % OPal); (**c**) OPal; (**d**) PMMA/OPal blend (5 wt % OPal).

The SEM micrographs of PMMA, OPal, poly(OPal-MMA), and the PMMA/OPal blend are shown in Figure 1. PMMA (Figure 1a) shows many spherical particles, while OPal (Figure 1c) presents a rod-like structure, with individual rods of about 200–500 nm in length and 30–80 nm in diameter. The poly(OPal-MMA) (Figure 1b) shows a net-shaped microporous structure. This structure could contribute to improving the absorption capacity of the electrolyte solution. The PMMA/OPal blend (Figure 1d) shows many spherical particles, and there are many rods of OPal between the particles of PMMA.

**Figure 2:**
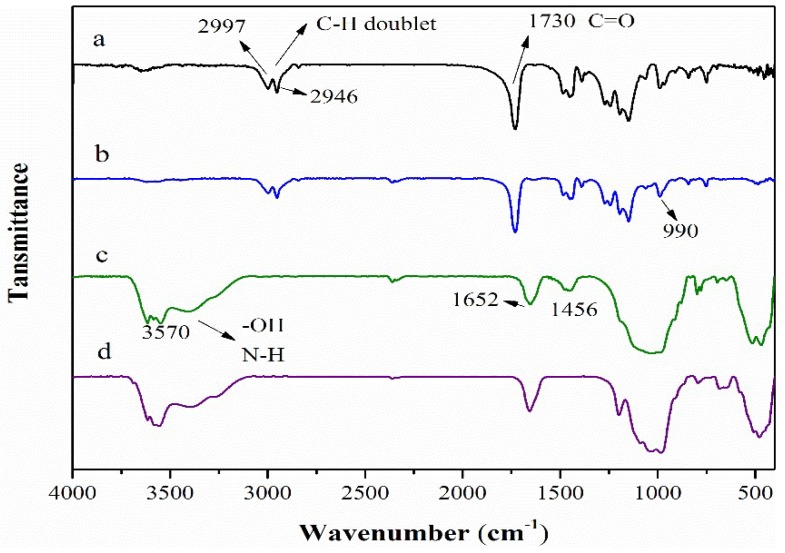
FTIR spectra of PMMA, poly(OPal-MMA), OPal, and Pal. (**a**) PMMA; (**b**) poly(OPal-MMA); (**c**) OPal; (**d**) Pal.

The FTIR curves of PMMA, poly(OPal-MMA), OPal, and Pal are shown in Figure 2. The spectrum of the Pal (Figure 2d) showed a band at 3600–3100 cm^−1^, attributed to the strong absorbance peak of OH group. The peak at 1652 cm^−1^ was ascribed to adsorbed water, and the peaks at 1200–990 cm^−1^ were related to the stretching vibration of Si–O. The spectrum of OPal (Figure 2c) showed additional peaks at 1456 cm^−1^ and 798 cm^−1^, which were related to the =C–H stretching vibrations. The spectrum of the pure PMMA (Figure 2a) showed a peak at 1730 cm^−1^, which was related to the stretching vibration of C=O. The characteristic bands at 2997 cm^−1^ and 2946 cm^−1^ corresponded to the stretching vibrations of –CH_3_. After the addition of the OPal, absorption bands of poly(OPal-MMA) (Figure 2b) appeared at 1050 cm^−1^ and 990 cm^−1^, due to the stretching Si–O vibration, which indicated that OPal has been introduced into polymers.

**Figure 3:**
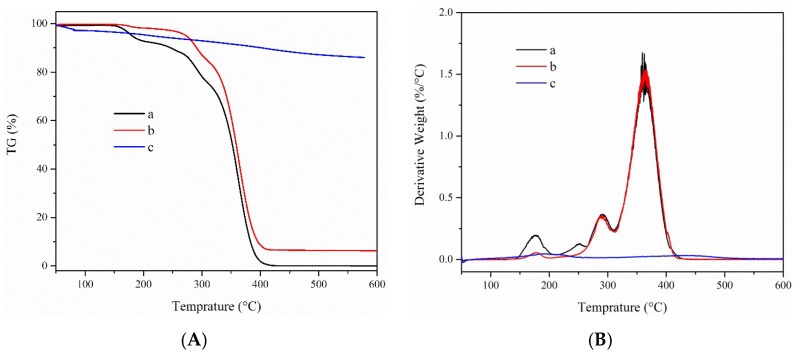
Thermo-gravimetry (TG) curves (**A**) and DTG curves (**B**) of PMMA, poly(OPal-MMA), and OPal. (a) PMMA; (b) poly(OPal-MMA); (c) OPal.

The thermal stability of the polymers was analyzed by thermo-gravimetry (TG) in a nitrogen atmosphere. Figure 3 shows the weight loss (TG) and weight loss rate (DTG) curves of PMMA, OPal, and poly(OPal-MMA). The DTG curve of PMMA showed four weight-loss peaks at 178 °C, 252 °C, 291 °C, and 364 °C. The weight loss of OPal increased with the increasing temperature, but no obvious weight loss peak was observed across the test temperature range, and its solid residue was about 86% at 600 °C. The poly(OPal-MMA) DTG curve showed peaks at 291 °C and 366 °C, and its solid residue was about 7.8% at 600 °C, while PMMA was completely degraded (solid residue was 0.03%). This indicated that the thermal stability of poly(OPal-MMA) was higher than that of PMMA.

### 3.2. Electrochemical Characterization of Polymers

The electrolyte uptake of poly(OPal-MMA) polymers with different OPal contents are shown in Table 1. The electrolyte uptake increased to a maximum with the increase of the OPal content, and then slightly decreased with the further increase of the OPal content. The highest electrolyte uptake of poly(OPal-MMA) was 263 wt % when the Pal weight ratio was 5%, while the uptake of the PMMA/OPal blend was 212 wt % at the same OPal concentration. The increase of the electrolyte uptake for poly(OPal-MMA) was mainly attributed to its net-shaped microporous structure.

Generally, the ionic conductivity of GPE is influenced by the amount of the liquid electrolyte uptake within the polymer matrix. The ionic conductivity of poly(OPal-MMA) with different OPal contents is also shown in Table 1. The ionic conductivity of poly(OPal-MMA) was greater than 1.0 × 10^−3^ S/cm. This value is a key factor that could determine whether the electrolyte has value for commercial application [30]. The highest ionic conductivity was 2.94 × 10^−3^ S/cm at 30 °C, which was observed when the OPal weight ratio was 5 wt %. The electrolyte uptake (263%) and ionic conductivity (2.94 × 10^−3^ S/cm) of poly(OPal-MMA) were far above the average observed for other PMMA/nanoparticle blends [19,20,21,22]. Generally, ionic conductivity is related to the concentration of carrier ions and their mobility. There are two conduction paths for the mechanism of ionic conduction, gel phase and pores full of liquid electrolyte [16]. Poly(OPal-MMA) had more pores than the PMMA/OPal blend, could absorb more liquid electrolyte, and formed a gel polymer electrolyte with better mobility and greater porosity. In the literature, PMMA/organophilic montmorillonite nanocomposite-based electrolytes attained a maximum value of 1.3 × 10^−3^ S/cm at 25 °C [22].

**Figure 4:**
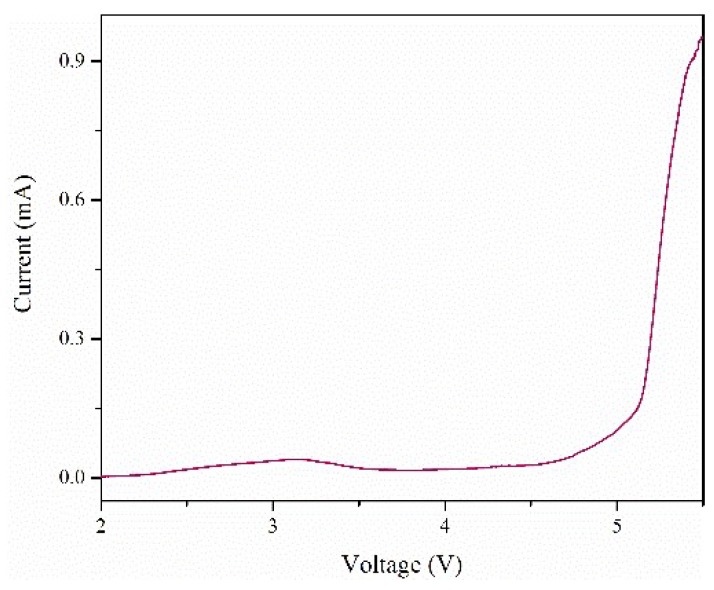
The electrochemical window of poly(OPal-MMA) (poly(OPal-MMA)-5).

The electrochemical window of the Li/poly(OPal-MMA) electrolyte/LiFePO_4_ cells is shown in Figure 4. Generally, the electrochemical window lies in the lithium salt complex and polymer host [31], and the decomposition voltage reflects the electrochemical window. In the process of the actual application a lithium battery, the work potential must reach 4.5 V (vs. Li^+^/Li). In this study, the electrochemical window of the poly(OPal-MMA) electrolyte was investigated using the linear sweep polarization method. The result showed that the electrochemical window of the poly(OPal-MMA) was higher than 4.5 V, which met the practical requirement of a LIB [5].

**Figure 5:**
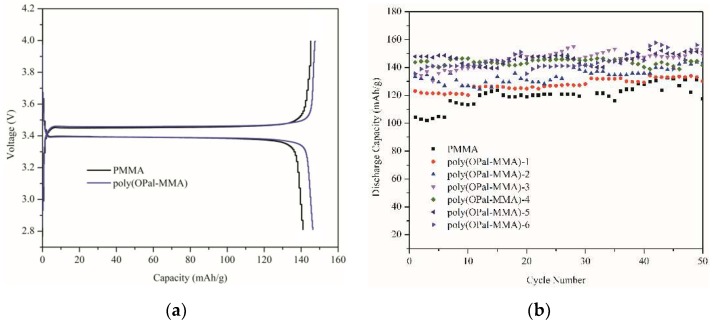
(**a**) Initial charge–discharge curves of the Li/poly(OPal-MMA) electrolyte/LiFePO_4_ cell and the Li/PMMA electrolyte/LiFePO_4_ cell at 0.1 °C, and (**b**) the cycle performance of cells with different polymer electrolytes at 0.5 °C.

In order to study the electrochemical stability of poly(OPal-MMA), the charge–discharge has been investigated, and the results are shown in Figure 5. Figure 5a presents the initial charge–discharge capacities of the Li/poly(OPal-MMA) electrolyte/LiFePO_4_ cell and the Li/PMMA electrolyte/LiFePO_4_ cell at a 54 μA/cm^2^ current density (corresponding to a C/10 rate). All the charge–discharge curves of the cells showed a flat plateau between 3.4 and 3.5 V, which is a typical characteristic of LiFePO_4_ [5]. Generally, the theoretical specific capacity of a LiFePO_4_ battery is 170 mAh/g [31,32]. Figure 5a also shows that the charge capacity of the Li/poly(OPal-MMA) electrolyte/LiFePO_4_ cell is greater than the discharge capacity. The irreversible capacity loss might be related to the delithiation process in the LiFePO_4_, the generation of the passivation layer at the lithium anode, and an increase in the internal resistance [33,34,35]. When the OPal concentration was 5%, the highest initial charge–discharge capacity of the cell was 147.57 mAh/g and 146.36 mAh/g, respectively, and the coulombic efficiency was 97.3%. Generally, battery coulombic efficiency is related to metallic lithium plating and its subsequent corrosion, as well as the formation and growth of a solid electrolyte interface film, and so on. [18]. Many studies have reported that PMMA can reduce the interface resistance and contact resistance between the electrolyte and the electrodes [16,18,34,36]. Cells that can absorb more electrolyte, and also have good interface compatibility between the polymer electrolyte and the electrodes will have an increased charge–discharge capacity. Figure 5b shows the cycle discharge capacities of the Li/poly(OPal-MMA) electrolyte/LiFePO_4_ cell. The discharge capacities of the cells with different separators were between 133 mAh/g to 147 mAh/g, and the capacity of the Li/poly(OPal-MMA) electrolyte/LiFePO_4_ cell was very stable. The capacity of the Li/poly(OPal-MMA)-5 electrolyte/LiFePO_4_ cell was 146.35 mAh/g after 50 cycles, and the decaying rate was as low as 0.02% per cycle.

## 4. Conclusions

Poly(OPal-MMA) was prepared by solution polymerization, using OPal and MMA as raw materials. The physical and electrochemical characteristics of poly(OPal-MMA) were investigated. The poly(OPal-MMA) copolymer showed a net-shaped microporous structure. Compared with pure PMMA, the charge–discharge capacity of poly(OPal-MMA) was increased. When the concentration of OPal was 5%, the highest electrolyte uptake and ionic conductivity of poly(OPal-MMA) were 263% and 2.94 × 10^−3^ S/cm at 30 °C, respectively. The Li/poly(OPal-MMA) electrolyte/LiFePO_4_ cell showed a wide electrochemical window at around 4.7 V. The initial capacity of the Li/poly(OPal-MMA) electrolyte/LiFePO_4_ cell was 146.36 mAh/g at 0.1 °C, and the capacity was 146.35 mAh/g after 50 cycles, with a capacity–decay rate of 0.02% per cycle.

## Figures and Tables

**Table 1:** The electrolyte uptake and ionic conductivity of poly(OPal-MMA).

Run	OPal Content (%)	Electrolyte Uptake (%)	Ionic Conductivity (× 10^−3^ S/cm)
poly(OPal-MMA)-1	1	196	1.39
poly(OPal-MMA)-2	2	225	1.05
poly(OPal-MMA)-3	3	248	2.10
poly(OPal-MMA)-4	4	257	2.77
poly(OPal-MMA)-5	5	263	2.94
poly(OPal-MMA)-6	6	248	2.62
PMMA/OPal blend	5	212	2.45

## References

[1] Lee Y., Lee J., Choi J., Yoon W., Kim D. (2013). Cycling characteristics of lithium powder polymer batteries assembled with composite gel polymer electrolytes and lithium powder anode. Adv. Funct. Mater..

[2] Liu W., Lee S., Lin D., Shi F., Wang S., Sendek A., Cui Y. (2017). Enhancing ionic conductivity in composite polymer electrolytes with well-aligned ceramic nanowires. Nat. Energy.

[3] Kim H., Periasamy P., Moon S. (2005). Electrochemical properties of the Li-ion polymer batteries with P(VdF-co-HFP)-based gel polymer electrolyte. J. Power Sources.

[4] Zhang H., Zhang P., Li Z., Sun M., Wu Y., Wu H. (2007). A novel sandwiched membrane as polymer electrolyte for lithium ion battery. Electrochem. Commun..

[5] Idris N., Rahman M., Wang J., Liu H. (2012). Microporous gel polymer electrolytes for lithium rechargeable battery application. J. Power Sources.

[6] Capiglia C., Saito Y., Yamamoto H., Kageyama H., Mustarelli P. (2000). Transport properties and microstructure of gel polymer electrolytes. Electrochim. Acta.

[7] Dissanayake M., Jayathissa R., Seneviratne V., Thotawatthage C., Senadeera G., Mellander B. (2014). Polymethylmethacrylate (PMMA) based quasi-solid electrolyte with binary iodide salt for efficiency enhancement in TiO_2_ based dye sensitized solar cells. Solid State Ionics.

[8] Gopalan A., Santhosh P., Manesh K., Nho J., Kim S., Hwang C., Lee K. (2008). Development of electrospun PVdF-PAN membrane-based polymer electrolytes for lithium batteries. J. Membr. Sci..

[9] Zhai W., Zhu H., Wang L., Liu X., Yang H. (2014). Study of PVDF-HFP/PMMA blended micro-porous gel polymer electrolyte incorporating ionic liquid [BMIM]BF_4_ for Lithium ion batteries. Electrochim. Acta..

[10] Zheng W., Wong S. (2003). Electrical conductivity and dielectric properties of PMMA/expanded graphite composites. Compos. Sci. Technol..

[11] Huang Y., Liu S., Yang W., Yu C. (2010). Surface roughness analysis and improvement of PMMA-based microfluidic chip chambers by CO_2_ laser cutting. Appl. Surf. Sci..

[12] Huang X., Brittain W. (2001). Synthesis and Characterization of PMMA Nanocomposites by Suspension and Emulsion Polymerization. Macromolecules.

[13] Zheng W., Wong S., Sue H. (2002). Transport behavior of PMMA expanded graphite nanocomposites. Polymer.

[14] Bohnke O., Frand G., Rezrazi M., Rousselot C., Truche C. (1993). Fast ion transport in new lithium electrolytes gelled with PMMA. 1. Influence of polymer concentration. Solid State Ionics.

[15] Bohnke O., Frand G., Rezrazi M., Rousselot C., Truche C. (1993). Fast ion transport in new lithium electrolytes gelled with PMMA. 2. Influence of lithium salt concentration. Solid State Ionics.

[16] Ma T., Cui Z., Wu Y., Qin S., Wang H., Yan F., Han N., Li J. (2013). Preparation of PVDF based blend microporous membranes for lithium ion batteries by thermally induced phase separation: I. Effect of PMMA on the membrane formation process and the properties. J. Membr. Sci..

[17] Ding Y., Zhang P., Long Z., Jiang Y., Xu F., Di W. (2009). The ionic conductivity and mechanical property of electrospun P(VdF-HFP)/PMMA membranes for lithium ion batteries. J. Membr. Sci..

[18] Choi N., Park J. (2001). New polymer electrolytes based on PVC-PMMA blend for plastic lithium-ion batteries. Electrochim. Acta.

[19] Park J., Cho J., Park W., Ryoo D., Yoon S., Kim J., Jeong Y., Lee S. (2010). Close-packed SiO_2_/poly(methyl methacrylate) binary nanoparticles-coated polyethylene separators for lithium-ion batteries. J. Power Sources.

[20] Cao J., Wang L., He X., Fang M., Gao J., Li J., Deng L., Chen H., Tian G., Wang J., Fan S. (2013). In situ prepared nano-crystalline TiO_2_–poly(methyl methacrylate) hybrid enhanced composite polymer electrolyte for Li-ion batteries. J. Mater. Chem. A.

[21] Liang B., Tang S., Jiang Q., Chen C., Chen X., Li S., Yan X. (2015). Preparation and characterization of PEO-PMMA polymer composite electrolytes doped with nano-Al_2_O_3_. Electrochim. Acta.

[22] Deka M., Kumar A. (2010). Enhanced electrical and electrochemical properties of PMMA–clay nanocomposite gel polymer electrolytes. Electrochim. Acta.

[23] Shubha N., Prasanth R., Hoon H., Srinivasan M. (2013). Dual phase polymer gel electrolyte based on non-woven poly(vinylidenefluoride-co-hexafluoropropylene)–layered clay nanocomposite fibrous membranes for lithium ion batteries. Mater. Res. Bull..

[24] Wang M., Dong S. (2007). Enhanced electrochemical properties of nanocomposite polymer electrolyte based on copolymer with exfoliated clays. J. Power Sources.

[25] Liu Y., Liu P., Su Z. (2008). Morphological characterization of attapulgite/poly(methyl methacrylate) particles prepared by soapless emulsion polymerization. Polym. Int..

[26] Yao P., Zhu B., Zhai H., Liao X., Zhu Y., Xu W., Cheng Q., Jayyosi C., Li Z., Zhu J., Myers K.M., Chen X., Yang Y. (2018). PVDF/Palygorskite nanowire composite electrolyte for 4 V rechargeable lithium batteries with high energy density. Nano Lett..

[27] Chen F., Lou D., Yang J., Zhong M. (2011). Mechanical and thermal properties of attapulgite clay reinforced polymethylmethacrylate nanocomposites. Polym. Advan. Technol..

[28] Xu H., Yang H., Zhang L., Ni Q., Gong F. (2015). Preparation and properties of polycarbonate nanocomposites using attapulgite grafted poly(methyl methacrylate) as a potential nanofiller. J. Appl. Polym. Sci..

[29] Kufian M., Aziz M., Shukur M., Rahim A., Ariffin N., Shuhaimi N., Majid S., Yahya R., Arof A. (2012). PMMA-LiBOB gel electrolyte for application in lithium ion batteries. Solid State Ionics.

[30] Manuel Stephan A. (2006). Review on gel polymer electrolytes for lithium batteries. Eur. Polym. J..

[31] Wu C., Lu M., Chuang H. (2005). PVdF-HFP/P123 hybrid with mesopores: A new matrix for high-conducting, low-leakage porous polymer electrolyte. Polymer.

[32] Padhi A., Nanjundaswamy K., Masquelier C., Okada S., Goodenough J. (1997). Effect of Structure on the Fe^3+^/Fe^2+^ Redox Couple in Iron Phosphates. J. Electrochem. Soc..

[33] Hu Q., Osswald S., Daniel R., Zhu Y., Wesel S., Ortiz L., Sadoway D. (2011). Graft copolymer-based lithium-ion battery for high-temperature operation. J. Power Sources.

[34] Peled E., Golodnitsky D., Ardel G. (1997). Advanced Model for Solid Electrolyte Interphase Electrodes in Liquid and Polymer Electrolytes. J. Electrochem. Soc..

[35] Yang F., Wang D., Zhao Y., Tsui K.-L., Bae S.J. (2018). A study of the relationship between coulombic efficiency and capacity degradation of commercial lithium-ion batteries. Energy.

[36] Zhang R., Chen Y., Montazami R. (2015). Ionic Liquid-Doped Gel Polymer Electrolyte for Flexible Lithium-Ion Polymer Batteries. Materials.

